# *In vitro* anti-inflammatory effects of AZD8999, a novel bifunctional muscarinic acetylcholine receptor antagonist /β2-adrenoceptor agonist (MABA) compound in neutrophils from COPD patients

**DOI:** 10.1371/journal.pone.0210188

**Published:** 2019-01-04

**Authors:** Javier Milara, Sonia Contreras, Alfredo de Diego, Marta Calbet, Mònica Aparici, Esteban Morcillo, Montserrat Miralpeix, Julio Cortijo

**Affiliations:** 1 Pharmacy Unit, Hospital Clínico Universitario, Valencia, Spain; 2 Health Research Institute INCLIVA, Valencia, Spain; 3 CIBERES, Health Institute Carlos III, Valencia, Spain; 4 Department of Pharmacology, Faculty of Medicine, University of Valencia, Valencia, Spain; 5 Respiratory Unit, University and Polytechnic La Fe Hospital, Valencia, Spain; 6 Almirall, R&D Centre, Barcelona, Spain; 7 Research and Teaching Unit, University General Hospital Consortium, Valencia, Spain; Imperial College London, UNITED KINGDOM

## Abstract

Recent evidence indicates that AZD8999 (LAS190792), a novel muscarinic acetylcholine receptor antagonist and β2-adrenoceptor agonist (MABA) in development for chronic respiratory diseases, induces potent and sustained relaxant effects in human bronchi by adressing both muscarinic acetylcholine receptors and β2-adrenoceptor. However, the anti-inflammatory effects of the AZD8999 monotherapy or in combination with corticosteroids are unknown. This study investigates the anti-inflammatory effects of AZD8999 in monotherapy and combined with fluticasone propionate in neutrophils from healthy and chronic obstructive pulmonary disease (COPD) patients. Peripheral blood neutrophils from healthy and COPD patients were incubated with AZD8999 and fluticasone propionate, individually or in combination, for 1h followed by lipopolysaccharide (LPS) stimulation for 6h. The IL-8, MMP9, IL-1β, and GM-CSF release was measured in cell culture supernatants. AZD8999 shows ~ 50% maximum inhibitory effect and similar potency inhibiting the released cytokines in neutrophils from healthy and COPD patients. However, while fluticasone propionate suppresses mediator release in neutrophils from healthy patients, COPD neutrophils are less sensitive. The combination of non-effective concentrations of AZD8999 (0.01nM) with non-effective concentrations of fluticasone propionate (0.1nM) shows synergistic anti-inflammatory effects. The studied mechanisms that may be involved in the synergistic anti-inflammatory effects of this combination include the increase of glucocorticoid receptor (GR)α and MKP1 expression, the induction of glucocorticoid response element (GRE) activation and the decrease of ERK1/2, P38 and GR-Ser226 phosphorylations compared with monotherapies. In summary, AZD8999 shows anti-inflammatory effects in neutrophils from COPD patients and induces synergistic anti-inflammatory effects when combined with fluticasone propionate, supporting the use of MABA/ICS combination therapy in COPD.

## Introduction

Chronic obstructive pulmonary disease (COPD) is a debilitating disease characterized by persistent airway and systemic inflammation, altering the lung architecture to promote airway obstruction. It is a major cause of morbidity and mortality with a high and increasing prevalence [1]. The current first-line maintenance treatment for COPD involves the use of bronchodilators, including long-acting muscarinic acetylcholine receptor antagonists (LAMAs) and long-acting β2-adrenoceptor agonists (LABAs). Although the inhaled corticosteroids (ICS) are the main anti-inflammatory therapy used in COPD, they have limited effects in improving lung function and have little or no effect on controlling the underlying chronic inflammation in COPD patients [2]. Therefore, current evidences are in favour of combined therapies. ICS + LABAs, LABA + LAMA or LAMA as monotherapy are common options for patients with increased risk of exacerbation with moderate symptoms. Triple therapy based on ICS in combination with LABAs and LAMAs is indicated in severe COPD at risk of exacerbations showing benefits in lung function, reduction of exacerbations and an improved quality of life [3]. However, a recent randomised clinical trial showed that ICS withdrawal did not increase the number of exacerbations in patients with severe COPD under LABA/LAMA/ICS triple therapy [4] which indicate the current controversy in the use of ICS.

Although evidence for drug combinations comes from clinical trials, scientific rationale for drug combinations can be explained by molecular interactions as previously outlined [5–8]. Recent evidence provided by our group indicates that LAMA can improve corticosteroid insensitivity in neutrophils from COPD patients inhibiting PI3Kδ and enhancing glucocorticoid response element transcription factor (GRE) with the consequent increased expression of corticosteroid anti-inflammatory related genes [9]. Although not studied in detail, these results indicate potential anti-inflammatory synergism between triple therapy based in LABA + LAMA + ICS.

Dual bronchodilator therapy based on inhaled LABA/LAMA is a common strategy in patients not fully controlled with monotherapies [10] and appears to be superior to LABA/ICS combination in some patients based on the FLAME clinical trial [11].

Bifunctional molecules with both muscarinic acetylcholine receptor antagonist and β2-adrenoceptor agonist activity (MABA) represent an alternative to use LAMA/LABA fixed dose combinations. MABA molecules show technical and pharmacokinetic advantages in the case of co-formulations of two bronchodilators with a third component [12].

AZD8999 (LAS190792) is a novel, inhaled MABA compound that has reached IIa clinical development as a maintenance therapy for the treatment of COPD (http://clinicaltrials.gov/show/NCT02059434). Recent evidence showed that AZD8999 has higher effects inhibiting bronchoconstriction in isolated human bronchi than tiotropium or olodaterol alone, and also in comparison with batefenterol, the furthest developed MABA [13], but whether AZD8999 has anti-inflammatory properties remain to be dissected. Since LAMA and LABA can improve the anti-inflammatory effects of corticosteroids, it seems reasonable to think that MABA could show synergistic anti-inflammatory properties when combined with corticosteroids in corticosteroid insensitive inflammatory cells such as neutrophils from COPD patients [6].

The aim of this study was to provide scientific evidence of the anti-inflammatory effects of AZD8999 in neutrophils from COPD patients as well as to analyze potential synergistic effects in combination with corticosteroids.

## Methods

All reagents were obtained from Sigma-Aldrich (St. Louis, MO, USA) unless otherwise stated.

### Patients

Peripheral blood neutrophils were obtained from COPD patients who were current smokers and from healthy non-smoking controls. The study population consisted of 22 patients with stable COPD, defined according to the 2013 GOLD guidelines [14], and 16 age-matched non-smoking healthy controls with normal lung function. The minimum washout period for systemic medications (roflumilast or corticosteroids) in stable COPD patients for sampling blood was 4 days, which avoided potential effects of chronic systemic medication on the results. All COPD patients were current smokers and had bronchitis. In the patients with stable COPD, there were no disease exacerbations within 2 weeks prior to sample collection. Routine lung function tests were performed to evaluate forced vital capacity (FVC), forced expiratory volume in one second (FEV1) and FEV1/FVC ratio using a Vitalograph® αIII spirometer (Vitalograph, Maids Moreton, UK). The clinical features of the study population are summarised in Table 1. This project was approved by the local Ethics Committee of the University General Hospital of Valencia, Spain. Written informed consent was obtained from each patient or volunteer before starting blood sampling and lung function testing.

**Table 1:** Clinical features of the study population.

	Healthy (n = 17)	Stable COPD (n = 22)
Age, yr	67.2 ± 3	65.4 ± 8
Sex (M/F)	13/4	16/6
Tobacco consumption, pack-yr	0	33.8±8*
FEV1, % pred	99 ± 4	52.1 ± 5*
FVC, % pred	97 ± 2	90.1 ± 8
FEV1/FVC %	98 ± 4	51.4 ± 4*
GOLD 1 (mild) patients, no.	0	0
GOLD 2 (moderate) patients, no.	0	4
GOLD 3 (severe) patients, no.	0	18
GOLD 4 (very severe) patients, no.	0	0
Receiving inhaled steroids, no.	0	18
Receiving theophyllines, no.	0	0
Receiving long-acting b2-agonist, no.	0	22
Receiving anticholinergics, no.	0	18
Total peripheral blood neutrophils	4.4 ± 0.2 x 10^9^/L	7.9 ± 1.4 x 10^9^/L*

COPD: chronic obstructive pulmonary disease; FEV1: forced expiratory volume in one second; FVC: forced vital capacity; Pack-yr = 1 year smoking 20 cigarettes-day. Data are mean ± SD.

* *P* < 0.05 related to Healthy subjects. COPD classification was assessed according with GOLD 2013 guidelines.

### Human neutrophil isolation and stimulation

Peripheral blood neutrophils were isolated from peripheral venous blood and cultured as previously described [9]. Neutrophils were adjusted to 500×10^3^ cells per well in 24-well plates and incubated in RPMI 1640 for 1 h at 37°C, 5% CO_2_. The cells were then left untreated or treated with AZD8999 (0.01 nM–1 μM; Almirall Laboratories, Barcelona, Spain), corticosteroid fluticasone propionate (0.1 nM–1 μM), or the PI3K inhibitor LY294002 (1 μM) for 1 h before they were stimulated with 1 μg of lipopolysaccharide (LPS)/ml. LPS 1μg/mL concentration was selected based on previous works using the same concentration and incubation times [15–17]. Furthermore, we obtained ~80% of maximal increase of IL-8 and MMP-9 release from neutrophils using LPS 1μg/mL concentration in preliminary studies.

The stimuli and drugs were incubated together with the cells for 6 h. Supernatants were collected and centrifuged at 120×g for 5 min. The cell-free supernatant was used to measure IL-8, metalloproteinase-9 (MMP9), granulocyte-macrophage colony-stimulating factor (GM-CSF) and IL-1β. Cellular extracts were used to measure mRNA expression after 6 h of cell stimulation. IL-8 levels were measured using a commercially available enzyme-linked immunosorbent assay kit for IL-8 (R&D Systems, Nottingham, UK) according to the manufacturer’s protocol. MMP9, GM-CSF and IL-1β were measured using LUMINEX technology, in accordance with the manufacturer’s protocol.

### Real-time RT-PCR

Total RNA and reverse transcription was performed as previously reported [9]. cDNA was amplified using specific primers together with probes predesigned by Applied Biosystems for macrophage migration inhibitory factor (MIF; cat. no. Hs00236988), mitogen-activated protein kinase phosphatase 1 (MKP-1; cat. no. Hs00610256), PI3K-δ (cat. no. Hs00192399), HDAC2 (cat. no. Hs00231032) and GRα (cat. no. Hs00353740_m1) genes in a 7900HT Fast Real-Time PCR system (Applied Biosystems) using Universal Master Mix (Applied Biosystems).

Expression of the target gene was calculated and expressed as 2^−ΔCt^ as described in detail previously [9].

### Glucocorticoid response element transfection assay

The Cignal GRE reporter assay kit (Qiagen, cat. no. 336841) was used to monitor the activity of glucocorticoid receptor-induced signal transduction pathways in cultured Beas2B epithelial cells as previously described in detail [9].

### Western blot

Western blot analysis was used to detect changes in p-ERK1/2, p-p38, GRα and phospho-serine 226-GR using a rabbit anti-human p-ERK1/2 (1:1,000) antibody (monoclonal antibody; Cell Signaling, Boston, MA, USA; cat. no. 4376S) normalised to total rabbit anti-human ERK1/2 (1:1,000) antibody (monoclonal antibody; Cell Signaling; cat. no. 4695); rabbit anti-human phospho-p38 (1:1,000) antibody (monoclonal antibody; Cell Signaling; cat. no. 4631) normalised to total rabbit anti-human p38 (1:1,000) antibody (monoclonal antibody; Cell Signaling; cat. no. 9212); total mouse anti-human β-actin (1:10,000) antibody (monoclonal antibody; cat. no. A1978; Sigma); or rabbit anti-human polyclonal phospho-GR-Ser226 (1:1,000) antibody (Novus Biologicals, Littleton, CO, USA; cat. no. NB100-92540), normalised to mouse anti-human monoclonal GRα (1;1,000) antibody (BD Biosciences, Franklin Lakes, NJ, USA; cat. no. 611227) as previously outlined in detail [9].

### PI3Kδ activity

To measure PI3Kδ activity, neutrophils from COPD patients were isolated and then incubated with AZD8999 (0.01nM and 1 nM) or LY294002 (1 μM) for 1 h. The cells were stimulated with LPS for 30 min and then centrifuged. Total protein was extracted and PI3Kδ activity was measured as described in detail previously [9].

### Analysis of results

The data were subjected to a non-parametric analysis, with p < 0.05 considered indicative of statistical significance. Data are expressed as the median with interquartile range of n experiments. When the comparisons concerned more than two groups, analysis of variance (Kruskal-Wallis test) was first performed. In the case of a global significant difference, between-group comparisons were assessed by the Dunn’s post-hoc test, which generalizes the Bonferroni adjustment procedure. The concentration of AZD8999 and fluticasone propionate producing 50% inhibition (IC_50_) was calculated from the concentration-response curves by nonlinear regression in neutrophils from healthy individuals and COPD patients using the GraphPad v.6 software.

## Results

### Anti-inflammatory effects of AZD8999 and fluticasone propionate in neutrophils from healthy and COPD patients

**Fig 1:**
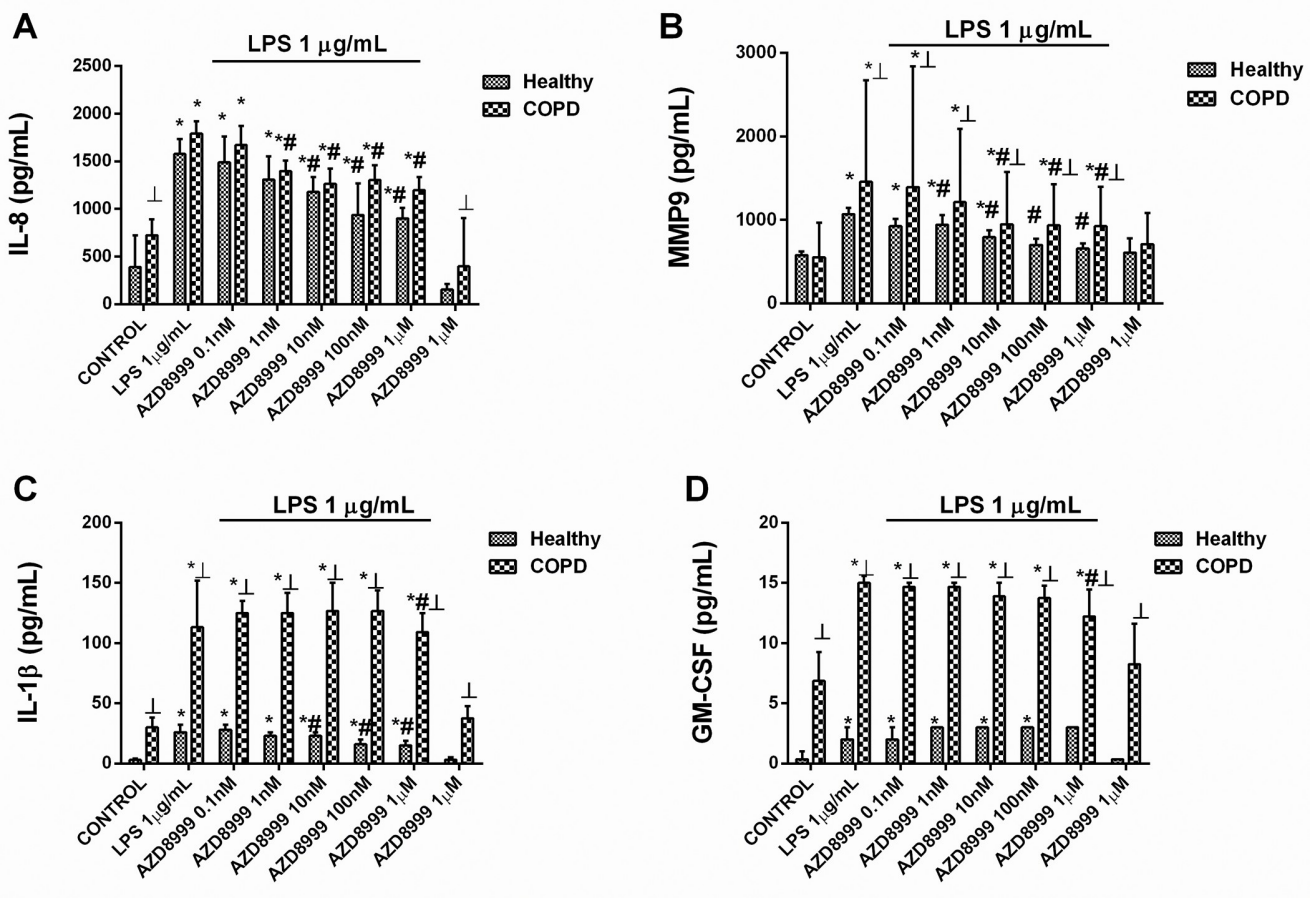
AZD8999 shows anti-inflammatory properties in human neutrophils. Concentration-dependent inhibition of lipopolysaccharide (LPS)-induced cytokines and MMP-9 release by AZD8999, from peripheral blood neutrophils of healthy controls and COPD patients. Neutrophils were preincubated with AZD8999 (0.1 nM–1 μM) for 1 h followed by cell stimulation with LPS (1 μg/ml) for 6 h. IL-8 (A), MMP9 (B), IL-1β (C) and GM-CSF (D) were determined in cell supernatants by ELISA. The results are expressed as the median with interquartile range (n = 3–4 each for cells from healthy controls and COPD patients in independent experiments with triplicate samples). Kruskal-Wallis test was followed by a Dunn’s post-hoc test. *p < 0.05 vs. control; #p < 0.05 vs. LPS; ┴p < 0.05 vs. cells from healthy patients.

**Fig 2:**
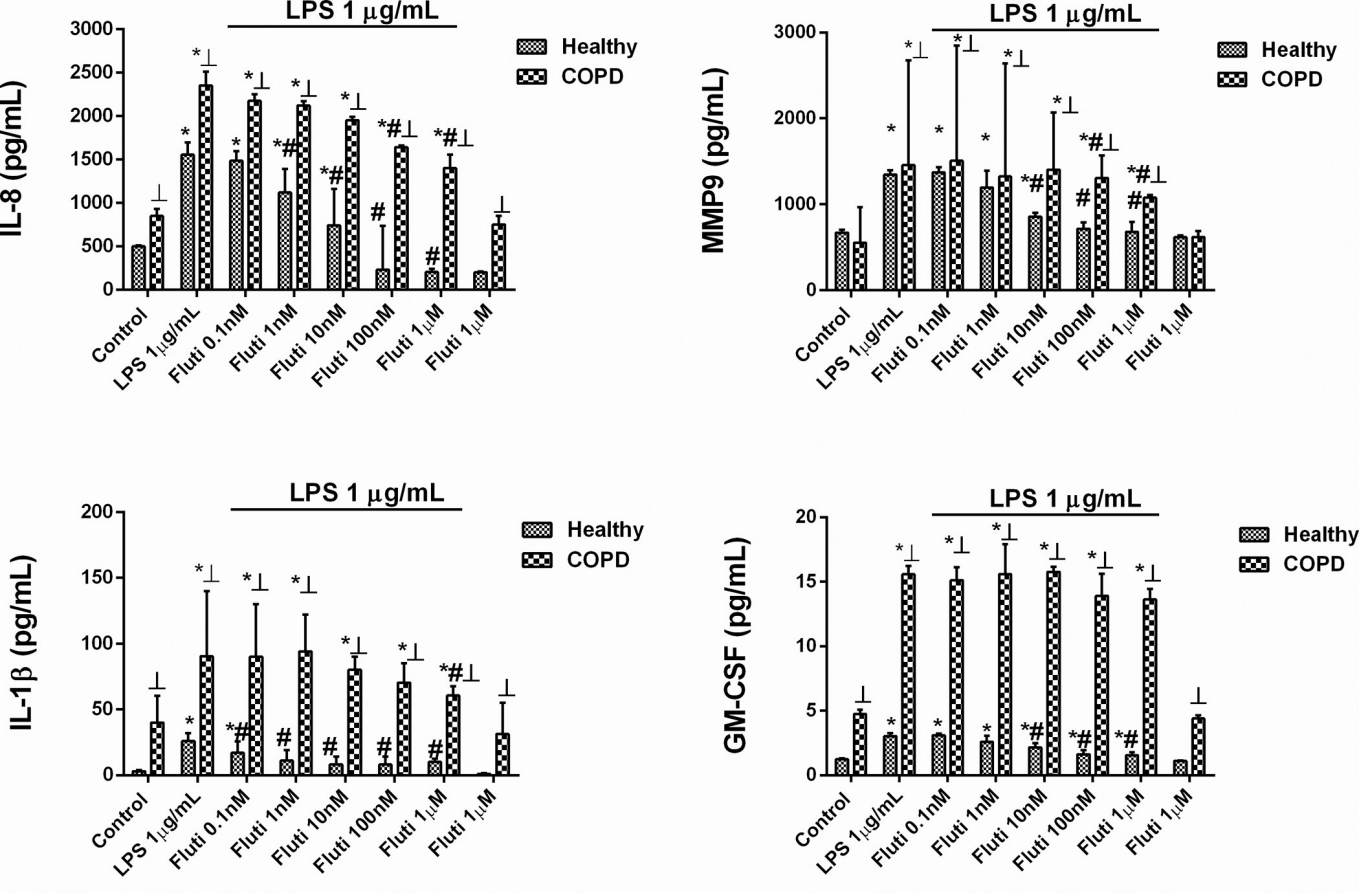
Fluticasone propionate shows impaired anti-inflammatory properties in human neutrophils from COPD patients. Concentration-dependent inhibition of lipopolysaccharide (LPS)-induced cytokines and MMP-9 release by fluticasone propionate (Fluti), from peripheral blood neutrophils of healthy controls and COPD patients. Neutrophils were preincubated with Fluti (0.1 nM–1 μM) for 1 h followed by cell stimulation with LPS (1 vg/ml) for 6 h. IL-8 (A), MMP9 (B), IL-1β (C) and GM-CSF (D) were determined in cell supernatants by ELISA. The results are expressed as the median with interquartile range (n = 3 each for cells from healthy controls and COPD patients in independent experiments with triplicate samples). Kruskal-Wallis test was followed by a Dunn’s post-hoc test. *p < 0.05 vs. control; #p < 0.05 vs. LPS; ┴p < 0.05 vs. cells from healthy patients.

AZD8999 concentration-dependently (0.1 nM–1 μM) inhibited the mediator secretion induced by LPS (1 μg/ml) similarly in both healthy and COPD neutrophils, showing the maximal % inhibition of ~ 50% for IL-8, ~70% for MMP9 and ~30% for IL-1β release (Fig 1A–1C and Table 2). GM-CSF inhibition was resistant to AZD8999 in neutrophils from healthy donors and inhibited by 73.4% in neutrophils from COPD patients (Fig 1D and Table 2). Unlike AZD8999, fluticasone propionate showed less potency and maximal % inhibition in neutrophils from COPD patients than in healthy neutrophils. Thus, the maximal % inhibition of fluticasone propionate was 117%, 95%, 75% and 82% for IL-8, MMP9, IL-1β and GM-CSF, respectively, in neutrophils from healthy subjects, and 57%, 60%, 61% and 19%, respectively, in neutrophils from COPD patients (Fig 2A–2D and Table 2).

**Table 2:** Maximal percentage of inhibition of IL-8, MMP-9, GM-CSF and IL-1β release from neutrophils of healthy subjects and COPD patients.

Cytokine	Healthy			COPD		
***IL-8***	Maximal % Inhibition	-log IC_50_	N	Maximal % Inhibition	-log IC_50_	N
AZD8999	46.5 ± 2.5	8.2 ± 0.2	5	50.5 ± 1.9	10.3± 0.2*	5
Fluti	117 ± 4.4#	7.8 ± 0.1	5	57.2 ± 3.3*	7.4± 0.14#	5
***MMP9***	Maximal % Inhibition	-log IC_50_	N	Maximal % Inhibition	-log IC_50_	N
AZD8999	77.7 ± 2.6	8.1 ± 0.1	5	62.9 ± 2	10 ± 0.1*	5
Fluti	95.3 ± 3.3#	8.5 ± 0.16	5	60 ± 3*	7.7 ± 0.12*#	5
***IL-1β***	Maximal % Inhibition	-log IC_50_	N	Maximal % Inhibition	-log IC_50_	N
AZD8999	39 ± 2.4	10.3 ± 0.3	3	27.5 ± 4	7 ± 1.8*	4
Fluti	75.6 ± 1.6#	10.4 ± 0.23	3	61.7 ± 3*	8.3 ± 0.17*	4
***GM-CSF***	Maximal % Inhibition	-log IC_50_	N	Maximal % Inhibition	-log IC_50_	N
AZD8999	0	ND	4	73.4 ± 5*	6.8 ± 0.15	4
Fluti	82.7 ± 3.6	8 ± 0.13	4	19.6 ± 3.3*	7.4 ± 0.4	4

Neutrophils were incubated with AZD8999 (0.1nM-1μM) or fluticasone (Fluti; 0.1nM-1μM) for 1h and stimulated with lipopolysaccharide (LPS 1μg/ml) for 6 h. The levels of different cytokines in the cell supernatant were determined and the maximal % of inhibitions were calculated. Values are mean ± SD of 3–5 independent donor experiments run in triplicate. IC_50_ values for half-maximum inhibition were calculated by nonlinear regression analysis.

*p < 0.05 vs Healthy values

#p<0.05 vs AZD8999 group.

ND: not determined.

### Effect of the combination of AZD8999 and fluticasone propionate on neutrophil response

**Fig 3:**
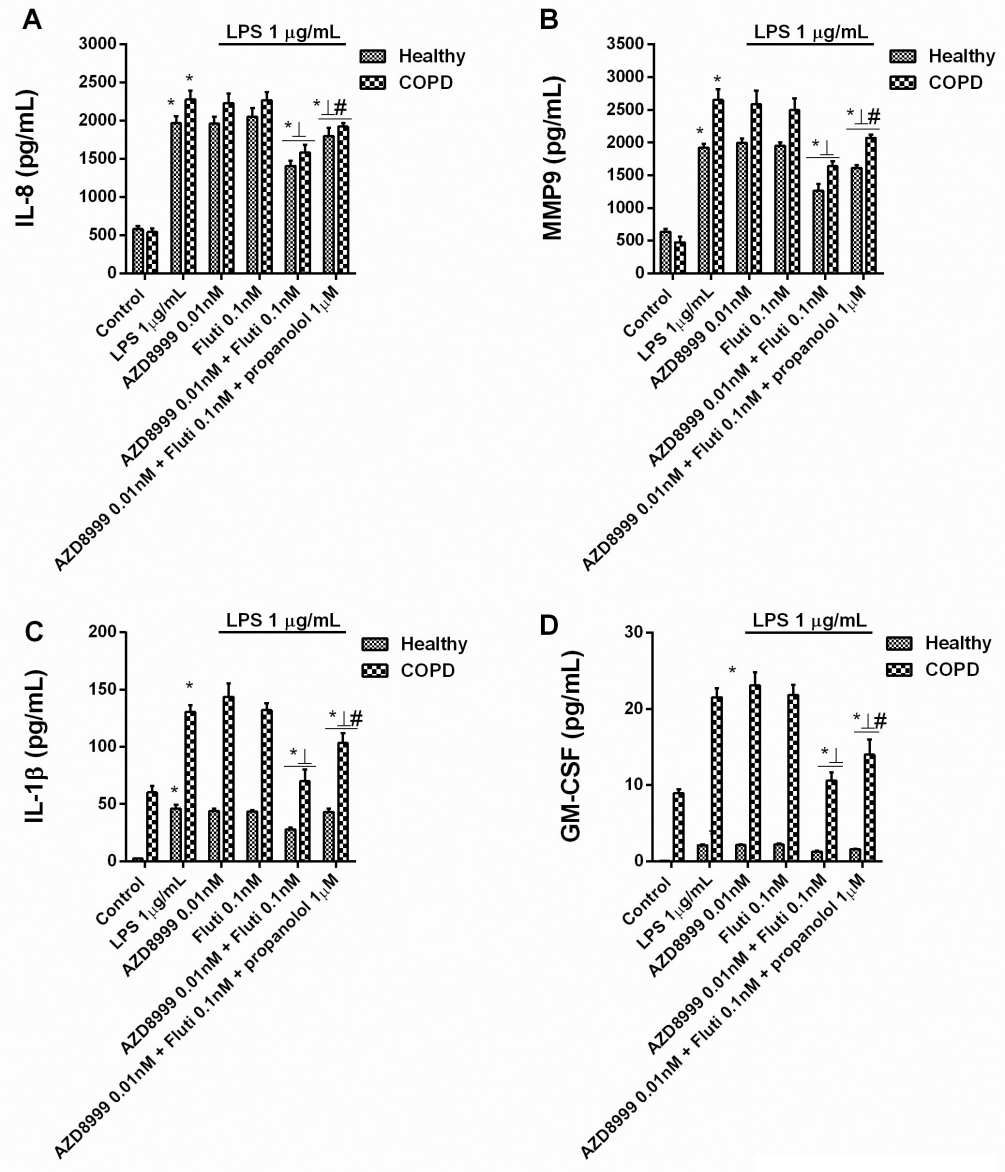
Effects of combined AZD8999 and fluticasone propionate on inflammatory mediators in neutrophils from healthy and COPD patients. Peripheral blood neutrophils from healthy controls and COPD patients were incubated with AZD8999, fluticasone propionate (Fluti) or both at non-effective concentrations for 1 h before they were stimulated with lipopolysaccharide (LPS) for 6 h. IL-8 (A), MMP9 (B), IL-1β (C) and GM-CSF (D) cytokine release was measured in cell supernatants. The results are expressed as the median with interquartile range (n = 3–5 each for cells from healthy controls and COPD patients in independent experiments with triplicate samples). Kruskal-Wallis test was followed by a Dunn’s post-hoc test. *p < 0.05 vs. control unstimulated cells; ⊥ p < 0.05 vs. monotherapy; #p < 0.05 vs. treatment without propranolol.

Whether the anti-inflammatory effects of AZD8999 and fluticasone propionate are synergistic was tested using non-effective concentrations of the drugs based on their concentration-dependent inhibitory curves (Figs 1 and 2). In peripheral blood neutrophils from healthy and COPD patients, AZD8999 at 0.01nM and fluticasone propionate at 0.1nM monotherapies showed no anti-inflammatory effects (Fig 3). When combined, AZD8999 at 0.01nM and fluticasone propionate at 0.1nM showed synergistic anti-inflammatory effects, resulting in nearly 50% inhibition of LPS-induced IL-8, MMP9, GM-CSF and IL-1β (Fig 3A–3D). The addition of β-adrenoceptor antagonist propranolol reduced the anti-inflammatory effect of the AZD8999 and fluticasone propionate combination to nearly 40%, suggesting the participation of both anti-muscarinic and β2-adrenoceptor agonism.

### Mechanisms implicated in the anti-inflammatory synergism observed with the combination of AZD8999 and fluticasone propionate

**Fig 4:**
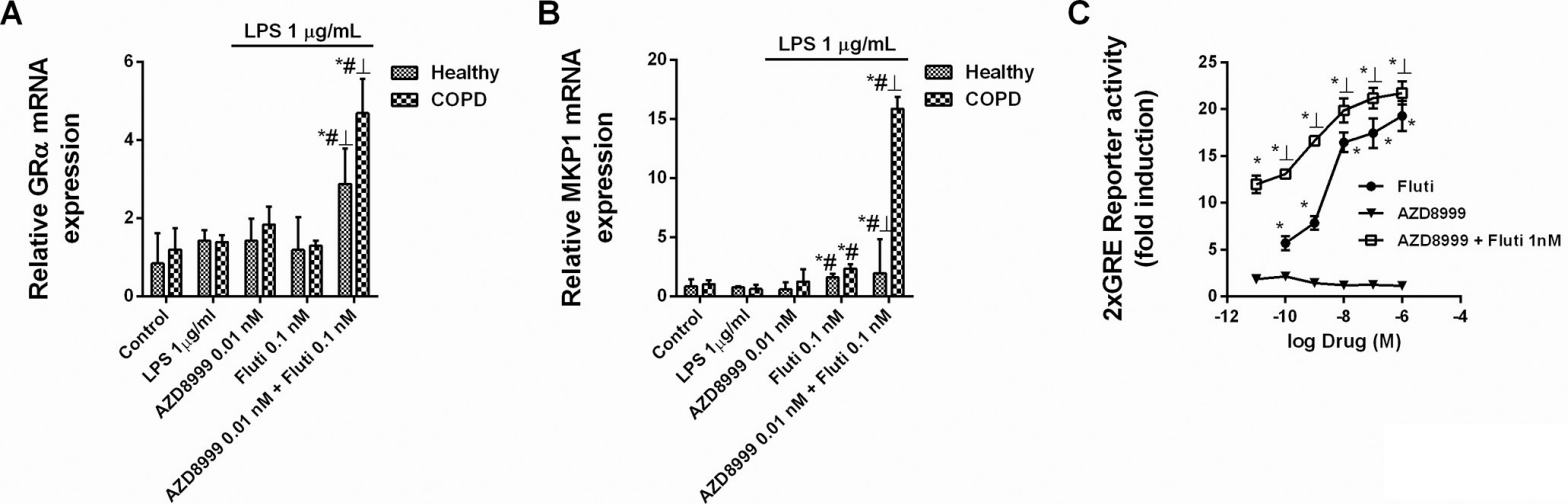
Effects of AZD8999, fluticasone propionate and combinations on corticosteroid signaling. (A, B) Human peripheral blood neutrophils isolated from healthy and COPD patients were incubated with AZD8999, fluticasone propionate (Fluti) or a combination thereof for 1 h, and stimulated with LPS for 6 h. Molecular corticosteroid modulators were quantified by RT-PCR using the 2^−ΔCt^ method, with expression of the housekeeping gene GAPDH serving as an internal control. The results are expressed as the median with interquartile range (n = 4 each for cells from healthy controls and COPD patients in independent experiments with triplicate samples). *p < 0.05 vs. control unstimulated cells; #p < 0.05 vs. LPS; ┴ p < 0.05 vs Fluti monotherapy. (C) Bronchial epithelial Beas2B cells were transfected with a GRE reporter gene and stimulated with different combinations of AZD8999 and Fluti. The results are expressed as the median with interquartile range of n = 3 independent experiments run in triplicate. Kruskal-Wallis test was followed by a Dunn’s post-hoc test. *p < 0.05 vs. AZD8999 monotherapy; ┴p < 0.05 vs. Fluti monotherapy.

**Fig 5:**
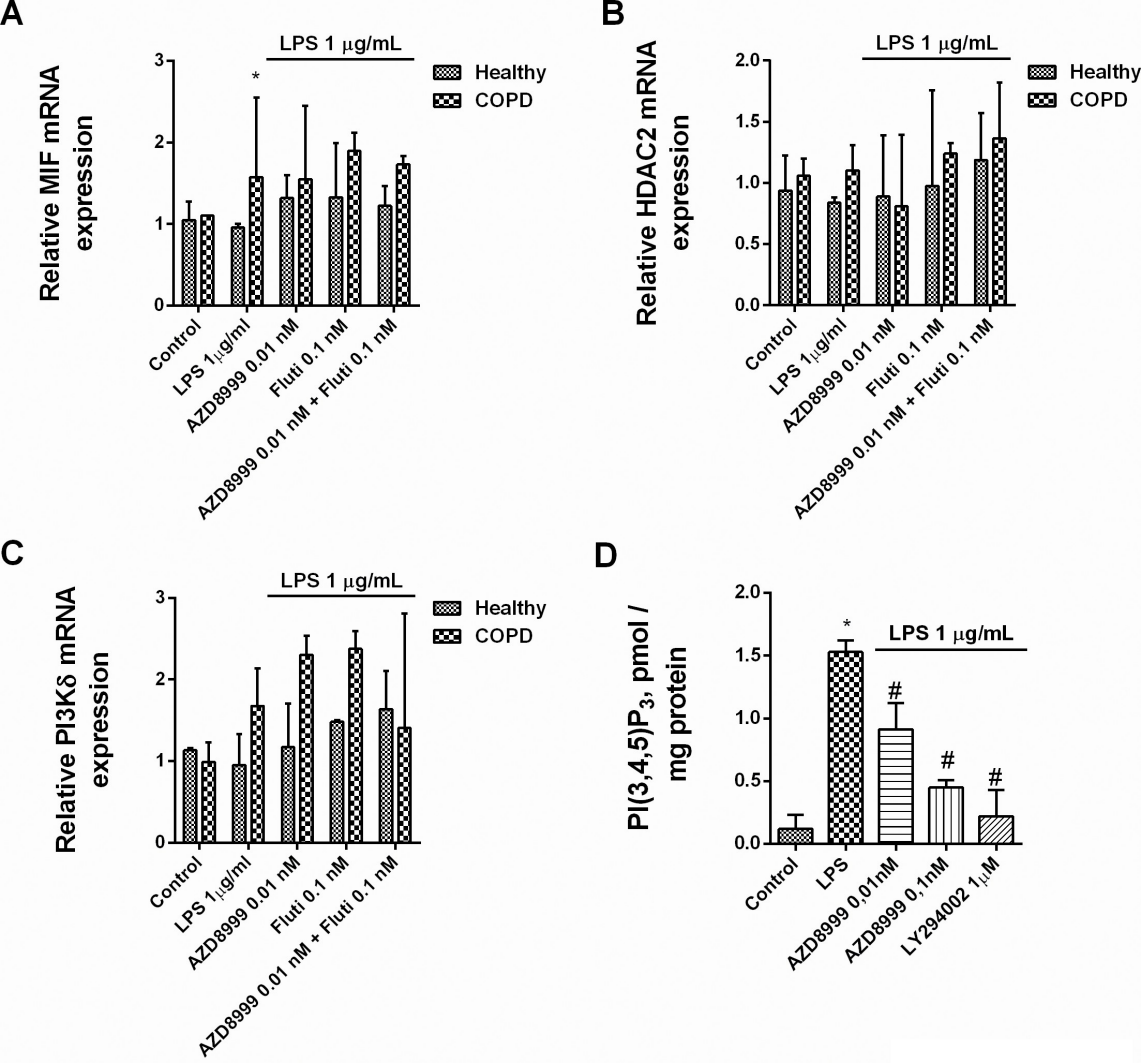
Effects of AZD8999, fluticasone propionate and combinations on lipopolysaccharide-induced corticosteroid modulators. (A-C) Human peripheral blood neutrophils isolated from healthy and COPD patients were incubated with AZD8999, fluticasone propionate (Fluti) or a combination thereof for 1 h, and stimulated with lipopolysaccharide (LPS) for 6 h. Molecular corticosteroid modulators were quantified by RT-PCR using the 2^−ΔCt^ method, with expression of the housekeeping gene GAPDH serving as an internal control. (D) PI3Kδ activity in human neutrophils from COPD patients. Peripheral blood neutrophils from COPD patients were incubated with AZD8999 or LY294002 for 1 h and stimulated with LPS for 30 min. The results are expressed as the median with interquartile range (n = 3 COPD cell populations in independent experiments run in triplicate). Kruskal-Wallis test was followed by a Dunn’s post-hoc test. *p < 0.05 vs. control unstimulated cells; #p < 0.05 vs. LPS-stimulated cells.

**Fig 6:**
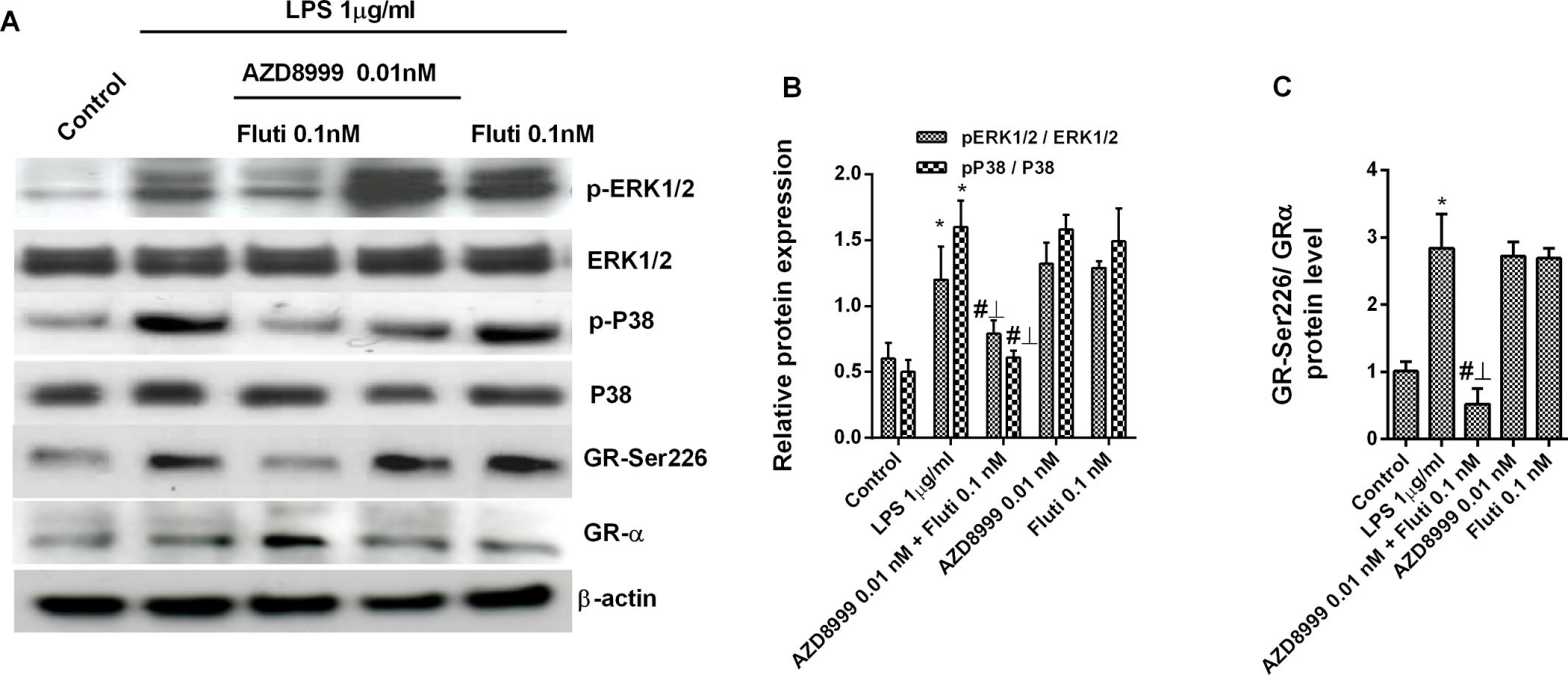
Combined AZD8999 and fluticasone propionate shows additive effects in inhibiting the lipopolysaccharide-induced phosphorylation of ERK1/2, p38 and GR-Ser226. Human peripheral blood neutrophils from COPD patients were incubated with AZD8999, fluticasone propionate (Fluti) or a combination thereof for 1 h and then stimulated with LPS during 30 min. Total protein was extracted for western blotting. The expression of p-ERK1/2, p-p38 and p-GR-Ser226 was determined as the ratio of the respective non-phosphorylated forms. Representative images are shown. Data are presented as the median with interquartile range (n = 3 COPD cell populations in independent experiments run in triplicate). Kruskal-Wallis test was followed by a Dunn’s post-hoc test: *p<0.05 vs. the control; #p<0.05 vs. LPS-stimulated cells. ⊥ p<0.05 *vs*. cells treated with drug monotherapy.

The combination of non-effective concentrations of AZD8999 and fluticasone propionate increased the gene expression of GRα and the anti-inflammatory corticosteroid inducible gene mitogen-activated protein kinase phosphatase 1 (MKP1) in neutrophils from healthy and COPD patients (Fig 4A and 4B). The activation of the GRE transcription factor was synergistically increased when fluticasone propionate was combined with AZD8999 in Beas2B epithelial cells (Fig 4C). The expression of the corticosteroid resistant markers MIF, HDAC2 and PI3Kδ was not affected by the AZD8999 and fluticasone propionate combination (Fig 5A–5C). In contrast, the activity of PI3Kδ was increased after LPS stimulation in neutrophils from COPD patients, supressed by the PI3K inhibitor LY294002 and significantly reduced by AZD8999 (Fig 5D). The stimulation of blood neutrophils from COPD patients with LPS increased the phosphorylation of ERK1/2, P38 and GR at serine 226 position (Fig 6A–6C). Monotherapy with AZD8999 and fluticasone at non-effective concentrations did not reduced protein phosphorylation. However, the combined therapy with AZD8999 and fluticasone propionate suppressed the protein phosphorylation (Fig 6A–6C).

## Discussion

This study shows for the first time the anti-inflammatory effects of a MABA compound, AZD8999, and the synergistic anti-inflammatory properties of AZD8999 and fluticasone propionate in neutrophils from COPD patients. While AZD8999 produced similar anti-inflammatory effects in neutrophils from healthy and COPD patients, fluticasone propionate suppresses inflammation in neutrophils from healthy subjects, but shows impaired anti-inflammatory effects in neutrophils from COPD patients. The combination of AZD8999 and fluticasone propionate shows synergistic anti-inflammatory effects and increases the impaired anti-inflammatory properties of the latter drug by a mechanism involving the increase of GRα expression, the activation of GRE transcription factor and the induction corticosteroid-dependent anti-inflammatory genes, such as MKP1, thus inhibiting the phosphorylation of ERK1/2, P38 and GR-Ser226. These results may be of potential clinical translational value pointing out the benefits of MABA in combination with ICS in severe COPD patients.

Several authors have proposed synergistic bronchodilation effects between LAMA and LABA since both share complementary mechanisms between the functional “cross-talk” of M2/M3 muscarinic and β2-adrenoceptors in airway smooth muscles [18]. MABA bifunctional compounds have the potential to deliver optimal bronchodilation after inhalation dosing via two validated mechanisms in one molecule as previously demonstrated with the most advanced MABA compounds such as GSK-961081 [19] and AZD8999 [20]. MABA offer a single pharmacokinetic profile for both pharmacologic activities, increasing potential synergy between the two mechanisms. In addition, MABA compounds offer a simplified triple therapy in one device.

Independently of the bronchodilator properties, LABA and LAMA have demonstrated modest anti-inflammatory properties in different cell types relevant in COPD [21, 22], in particular, neutrophils [23]. Both, LAMA and, in a lesser extent, LABA have shown anti-inflammatory properties in neutrophils from COPD patients [9, 24] which represents a functional expression of non-neuronal muscarinic acetylcholine receptor and β2-adrenoceptors, as previously we outlined [9]. In this study, we have shown that the MABA compound AZD8999 inhibits the release of relevant cytokines and metalloproteinases involved in COPD, confirming its anti-inflammatory role. The inhibitory potency of AZD8999 in the LPS-stimulated neutrophil assay is superior to that observed for the muscarinic acetylcholine receptor antagonist aclidinium or the β-adrenoceptor agonists salmeterol and formoterol, as previously described [9]. These results suggest additive effects between both pharmacology activities of the MABA compound.

As expected, fluticasone propionate shows more effect than AZD8999 inhibiting inflammation in neutrophils from healthy subjects. However, fluticasone propionate shows impaired anti-inflammatory effects in neutrophils from COPD patients, as it has been previously described [6], that were comparable to the effects of AZD8999. The loss of anti-inflammatory efficacy of corticosteroids in COPD patients has been revised extensively [25]. In fact, the use of corticosteroids as monotherapy in COPD is not indicated. Instead, the combination of ICS with LABAs or triple therapy including ICS + LABA and LAMA is broadly used in mild to very severe COPD when exacerbations occurred [3]. The mechanisms that can explain the benefits of combined therapy include the corticosteroid-induced β2-adrenoceptor (β2AR) expression, resulting in increased expression of cell surface β2AR and the inhibition of un-coupled β2AR-G_i_ protein [26], which prevents the desensitization of β2AR induced by chronic inflammation. In addition, there is now increasing evidence that β2-adrenoceptor agonists may affect GR function enhancing the anti-inflammatory effects of corticosteroids. In this line, formoterol activates serine/threonine protein phosphatase 2A (PP2A) that inhibits GR phosphorylation at serine 226 allowing the GR nuclear translocation [27]. LABA can also reverse corticosteroid insensitivity, inhibiting phosphoinositide-3-kinase delta (PI3Kδ) and allowing the proper function of histone deacetylase-2 (HDAC2) and GRα [7].

Recently, our group has demonstrated that anti-muscarinic drugs, such as aclidinium bromide, increase the anti-inflammatory effects of corticosteroids inhibiting GR phosphorylation at serine 226, increasing GRE activity and the anti-inflammatory gene expression in neutrophils from COPD patients [9]. These results indicate common mechanisms of muscarinic acetylcholine receptor antagonits and β2-adrenoceptor agonists improving the anti-inflammatory effects of corticosteroids, which could support the clinical efficacy of triple therapy. In line with this hypothesis, in this study we describe an anti-inflammatory synergism between AZD8999 and fluticasone propionate. AZD8999 has shown an IC_50_ potency of 1nM inhibiting human bronchi contraction in electrical field stimulated (EFS) preparations [20]. In this work, AZD8999 at non-effective 0.01nM concentration shows synergistic anti-inflammatory effects with 0.1nM non-effective fluticasone propionate concentration, inhibiting all the pro-inflammatory mediators tested, in both healthy and COPD neutrophils. The addition of the β-adrenoceptor antagonist propranolol reduced about 50% the inhibitory effect of AZD8999 and fluticasone propionate combination which demonstrates bifunctional activity of AZD8999. Previous *in vitro* experiments indicate predominant β2AR activation over the muscarinic acetylcholine receptor antagonism effects for the AZD8999 MABA [20]. In human neutrophils from COPD patients, the anti-inflammatory effects of aclidinium bromide were higher than that observed for salmeterol or formoterol suggesting a predominant role for non-neuronal cholinergic system activity [9]. Therefore, although AZD8999 shows predominant β2AR activation, the low 50% inhibition of propranolol could be explained by the higher non-neuronal cholinergic activity in COPD neutrophils.

Synergistic mechanism between AZD8999 and fluticasone could be explained, almost in part, by the increase of GRα expression, the increase of GRE activation, the corresponding induction of the anti-inflammatory gene MKP1 and the inhibition of phosphorylation of ERK1/2, P38 and GR-Ser226. Previous reports have demonstrated that corticosteroids in combination with β2AR agonists can increase GRα expression [24]. However, when combined with fluticasone propionate, the muscarinic acetylcholine receptor antagonist aclidinium bromide does not increase GRα expression [9]. Therefore, AZD8999 increases the expression of GRα when combined with fluticasone propionate probably due to the β2AR agonist component.

In conditions of chronic inflammation and oxidative stress, as occurs in COPD, several activated intracellular proteins such as p-ERK1/2 and p-P38 can phosphorylate GRα at specific residues such as serine 226 [27, 28]. Phosphorylation at GR-Ser226 inhibits the proper GRα nuclear translocation thus impairing corticosteroid anti-inflammatory effects. In this work we show that AZD8999 in combination with fluticasone propionate at non-effective concentrations suppresses GR-Ser226 phosphorylation induced by LPS and allows synergistic activation of GRE and consequently the increase of the anti-inflammatory gene MKP1. Similar results have been published for the corticosteroid/β2AR agonist [29] and corticosteroid/ muscarinic acetylcholine receptor antagonist combinations [9] supporting our findings. However, the concentrations used in previous studies were 100nM of salmeterol [29], 10nM aclidinium [9] and 1nM of fluticasone propionate respectively, higher than those used in the present work (AZD8999 0.01nM and fluticasone propionate 0.1nM combination) suggesting synergistic anti-inflammatory properties of MABA/corticosteroid combination.

MKP1 has the function to inhibit the phosphorylation of several MAP kinases such as ERK1/2 and P38, thus blocking their activation and the consequent increase of pro-inflammatory gene expression. In this work, AZD8999/fluticasone propionate combination shows synergistic inhibition of LPS-induced ERK1/2 and P38 phosphorylation which may be a consequence of the induction of MKP1 expression as previously outlined with corticosteroid/LABA and corticosteroid/LAMA *in vitro* therapy [9, 24].

The loss of HDAC2 activity is perhaps the most studied corticosteroid resistant molecular pathway [30]. Oxidative stress generated by cigarette smoke and chronic inflammation promotes the activation of PI3Kδ that inactivates HDAC2 [31]. The inactivation of HDAC2 impede the GRα/NF-κB complex effective suppression of activated inflammatory genes within the nucleus [25]. Previous works have shown that LABA and LAMA monotherapies when combined with corticosteroids can reduce the activation of PI3Kδ thus enhancing the anti-inflammatory effects of corticosteroids [7, 9]. In this work, AZD8999 inhibits the LPS induced PI3Kδ activation which may reinforce the molecular pathways of MABA/corticosteroid synergism.

The use of peripheral blood neutrophils instead of sputum neutrophils could represent a limitation of this study since tissue neutrophils may have different behaviour under inflammatory conditions. However, previous works showed similar results in neutrophils from peripheral blood or airway sputum from COPD patients reinforcing the value of our findings [9]. Although neutrophils play a key role in COPD pathogenesis [32], there are a broad number of inflammatory cells and molecular mediators that participate in lung inflammation and remodelling of COPD patients which represent a limitation of this *in vitro* study. It is also important to note that neutrophils used in this study as healthy controls were from non-smokers subjects instead smoker healthy subjects who have been shown impaired anti-inflammatory effects to corticosteroids [33], and may therefore represent a limitation of this study.

In summary, this is the first report showing the anti-inflammatory effects of the dual MABA compound, AZD8999, and the potential anti-inflammatory synergism between MABA and corticosteroids which may be of potential value to explain, almost in part, possible benefits of MABA/ICS combination therapy in severe COPD patients.

## Supporting information

S1 File Raw data. (XLSX)
